# Embodied Organization of *Octopus vulgaris* Morphology, Vision, and Locomotion

**DOI:** 10.3389/fphys.2017.00164

**Published:** 2017-03-28

**Authors:** Guy Levy, Binyamin Hochner

**Affiliations:** Department of Neurobiology, Silberman Institute of Life Sciences, Hebrew UniversityJerusalem, Israel

**Keywords:** octopus vulgaris, motor control, soft bodied animal, embodiemnt, Mollusca

The rich motor behavior of *Octopus vulgaris* is an outstanding biological example of motor control in a soft-bodied animal. The flexible hyper-redundant arms of the octopus endow it with high maneuverability but also place a great burden on its control system. The main difficulty in using the arms for precise goal-directed movements and coordinated locomotion is the problems of interfacing the incoming sensory information with the issuing of the proper motor commands. Skeletal animals evolved a solution for this interfacing difficulty by employing central “representation maps” that represent the sensory and the motor information in an organization that maintains the spatial relationships of the body morphology (somatotopic representation), yet the relative size of each body part reflects the number of sensory receptors and the number of muscle groups in each of the body parts. Therefore, in our brain, this brain organization resembles a topography of a “little man”—homunculus in Latin. The implication of such topological organization is that in the central brain, (e.g., in our motor and sensory cortices) the sensory and motor activities are represented in “body parts coordinates.” This representation format likely serves as a useful “reference table” for the brain to compute feedforward motor commands for motor interaction with the external world. This computational mechanism is feasible because the number of body parts and their dynamic locations with respect to each other is constrained by the limited number of joints and the fixed configuration of the skeleton which limits the number of controlled parameters (i.e., degrees of freedom, DOFs) needed to be computed for the execution of specific movements. Implementing in the octopus a motor control mechanism that is similarly based on body parts representation would be ineffective because of the lack of fixed spatial relationships between the flexible body parts that would require an enormous computational power to calculate the feedforward commands that are needed to control the enormous number of DOFs that are required for computing the coordinated interaction of eight long and flexible arms with the external world. Indeed, the body of the octopus is not represented somatotopically in the higher motor centers (the basal lobes) in the octopus brain (Zullo et al., 2009) and as we describe below, the evolved control algorithms of the arms in goal directed movement and locomotion highlights control strategies that seem to overcome the need for central representation of the body.

Previous and more recent results suggest that the solution for this difficulty has evolved through “embodied evolution” of the octopus unique morphology to enable the nervous system to employ special motor-control strategies that alleviate the need to rely on central body parts representation (reviews: Zullo and Hochner, 2011; Hochner, 2012, 2013).

Here, we first give a short account of the unique mechanisms that have evolved to simplify the control in goal-directed movements, and then present new surprising results that suggest a control mechanism for coordinating the flexible appendages during locomotion and show how vision of *Octopus vulgaris* is embodied in this novel locomotion control mechanism.

## Control of goal-directed movements

The reaching movement, as first example, is controlled by a motor program that does not depend on body-part coordinates and is essentially a stereotypical movement combination of several motor primitives; the arm is extended toward the target by propagating a bend along it and independently controlling elongation of the arm segment that is proximal to the propagating bend (Gutfreund et al., 1996, 1998; Hanassy et al., 2015). This control strategy reduces the number of DOFs involved in the central control of reaching to only three or four: two DOFs are needed for controlling the direction of the base of the arm, one for the propagation of the stiffening wave that pushes the passive bend forward, and possibly another DOF for controlling the elongation and straightening of the arm. Amputated arms can generate the same typical arm extension when the exposed axonal tract of the arm nerve cord is given a short train of electrical stimulations, indicating that the motor program for generating arm extension is embedded in the peripheral neuromuscular system of the arms (Sumbre et al., 2001).

In goal-directed fetching movements the octopus brings food precisely to its mouth. To do this, the long, flexible arm is “reshaped” into an “articulated,” skeletal-like structure of three segments with the proximal and medial segments having similar length and the food is held by the distal segment using a group of suckers (serving as a hand). The food is brought to the mouth by rotating the pseudo-elbow situated between the proximal and media segments. As in our arms, the equal segment lengths simplify the precise reaching of the distal segment to the mouth that is located, in the octopus, at the center of a circle created by the bases of the arms. But, in sharp contrast to articulated skeletal appendages, pseudo-articulations in the octopus are dynamic and are reshaped for each fetching movement and are adjusted according to the holding position of the target along the arm. At first, this seems somewhat puzzling, as it is hard to perceive a simple way for the central nervous system to coordinate such a dynamic structure but remarkably, the octopus uses the arm itself for calculating the site of the pseudo elbow. After contact with the target is made, two waves of muscle activation start traveling toward each other—one propagates from the site of contact with the target proximally along the arm, and the other propagates from the base of the arm distally along it. The elbow is formed where the two waves collide (Sumbre et al., 2006).

The motor programs for these two goal-directed movements are embedded in the neuromuscular system of the arm (Sumbre et al., 2001, 2006). This notion is supported by newer findings showing that the motor programs are represented in the higher motor centers of the octopus brain (Zullo et al., 2009). So at least for some movements, the higher motor centers in the octopus central brain, in contrast to skeletal animals, are involved only in the activation and scaling of peripheral programs and in adjusting the movements according to relevant visual and tactile information by controlling only few DOFs that are involved in their execution.

These results show very clearly that special evolutionary solutions have evolved to cope with the complex motor control problems of goal directed movements in hyper-redundant appendages.

## Control of arm coordination in locomotion

For all of us the way *Octopus vulgaris* is moving swiftly around in the aquarium or in nature seems very elegant and effortless. This should amaze us because it is not simple to perceive a control system that can mediate such locomotion capabilities in a hyper-redundant body that lacks a skeleton. Indeed, *Octopus vulgaris* appears to have evolved unique control mechanisms that enable it to coordinate its eight arms efficiently during various forms of locomotion. The main difficulty in controlling locomotion with the long and flexible appendages of the octopus arises from the fact that they lack any structural constrain. Thus, no type of feedforward control mechanism can be easily implemented in its locomotion (unless, in theory, a supercomputational power could have been integrated in the control). This sharply contrasts the requirements of computational power necessary to control skeletal appendages, where a small number of joints limit the interactions with the environment to a small number of DOFs, making the control of locomotion feasible with repeated rhythmical patterns of motor output generated by rather simple central pattern generators (CPGs). This is a universal control mechanism found in all types of locomotion throughout the animal kingdom.

**Figure 1:**
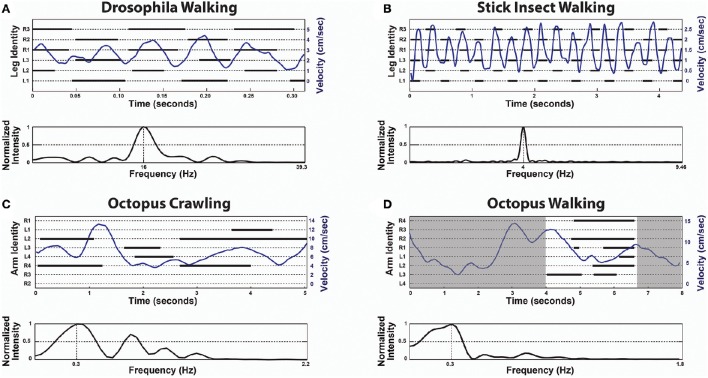
**In contrast to the universal role of CPGs in locomotion, octopus locomotion involves ***ad hoc*** recruitment of the arms interacting with the environment**. Upper panels are Stepping Records (black) with the body's instantaneous velocity superimposed (blue) and lower panels give the spectrum of frequencies of the respective velocity extracted by Fast Fourier Transform (FFT). In the upper panel of **(D)** only the time interval between 4 s and about 6.6 s was analyzed (white area) because in the rest of the time there was obscuring of some of the arms. **(A)** Drosophila Walking, originally adapted from Mendes et al. (2013). **(B)** Stick insect (*Carausius morosus*) Walking, originally adapted from Graham (1972). **(C)** Octopus Crawling. **(D)** Octopus Walking. Note the lack of temporal pattern in **(C,D)**. The extracted frequencies of octopus crawling and walking merely reflect the window sizes (for example, the frequency of 0.3 Hz in **(C)** means a cycle every 3.3 s, but the entire movement lasts only 6 s). In contrast, the extracted frequencies of Drosophila and stick insect walking each shows a single prominent characteristic frequency (reflecting the underlying CPGs rhythmicity).

The first indication that the octopus is a unique exception and lacks CPGs in locomotion control was found by studying arm coordination during crawling (Levy et al., 2015). The octopus crawls by making moment-to-moment *ad hoc* decisions; essentially choosing which of its arm(s) to recruit for pushing the body. A group of suckers on the chosen arm(s) adheres to the substrate, giving an anchoring point for a stereotypical elongation of a proximal segment to generate the thrust. The moment-to-moment direction of crawling is determined by a vectorial summation of the pushing directions of the active arms, where each arm has a single predefined pushing direction that is determined by its position around the body. This calculation is simple because the arms are organized in a radial symmetry around the body and the active arms at each moment in time apply virtually equal pushing forces. As shown in Figure 1 there is no apparent order in the octopus arm stepping records (C) and Fast Fourier Transform (FFT) analysis of instantaneous crawling velocity did not reveal any characteristic frequencies that would indicate of the presence of a rhythmical CPG, as clearly evident in a similar analysis of insect walking (Figures 1A,B, originally adapted from Mendes et al., 2013 and from Graham, 1972, respectively).

We are now investigating the mechanism of arm coordination during several octopus locomotion maneuvers. Again, the results are surprising and further indicative of the existence of unique locomotion control mechanisms.

**Figure 2:**
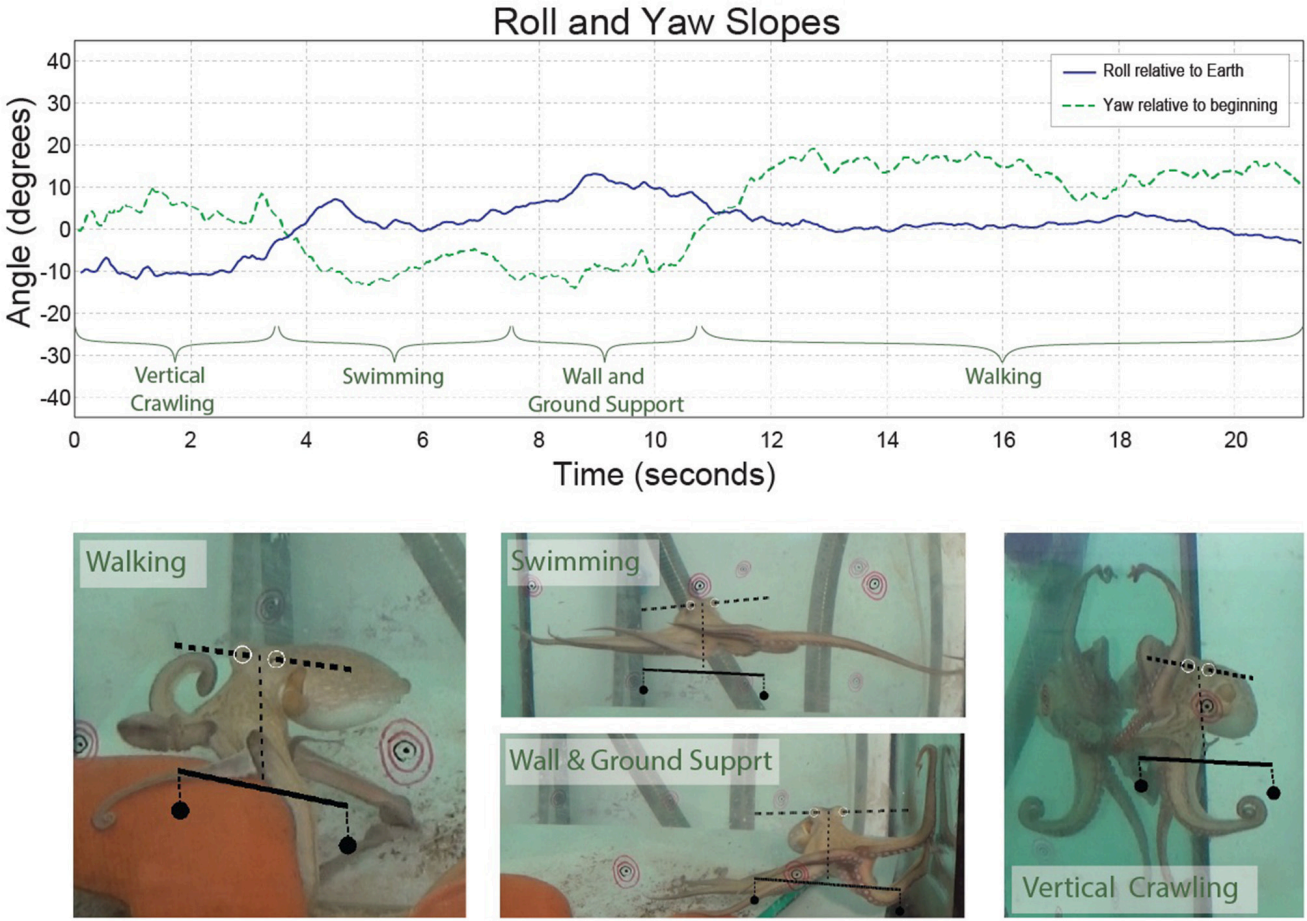
**The slope between the eyes during behavior (roll–blue)**. The yaw slope (green dashed line) was added for comparison. The time through which the octopus showed each type of locomotion is marked on the plot and video images. The slope of the axis that runs between the eyes (dashed line) and the scale below it (continuous line) are shown on the video and images. The scale shows what would be zero-degree orientation relative to Earth. The concentric black and red circles were physically on the aquarium and served for calibration of the three cameras to reconstruct the 3D position of the eyes.

**Figure 3:**
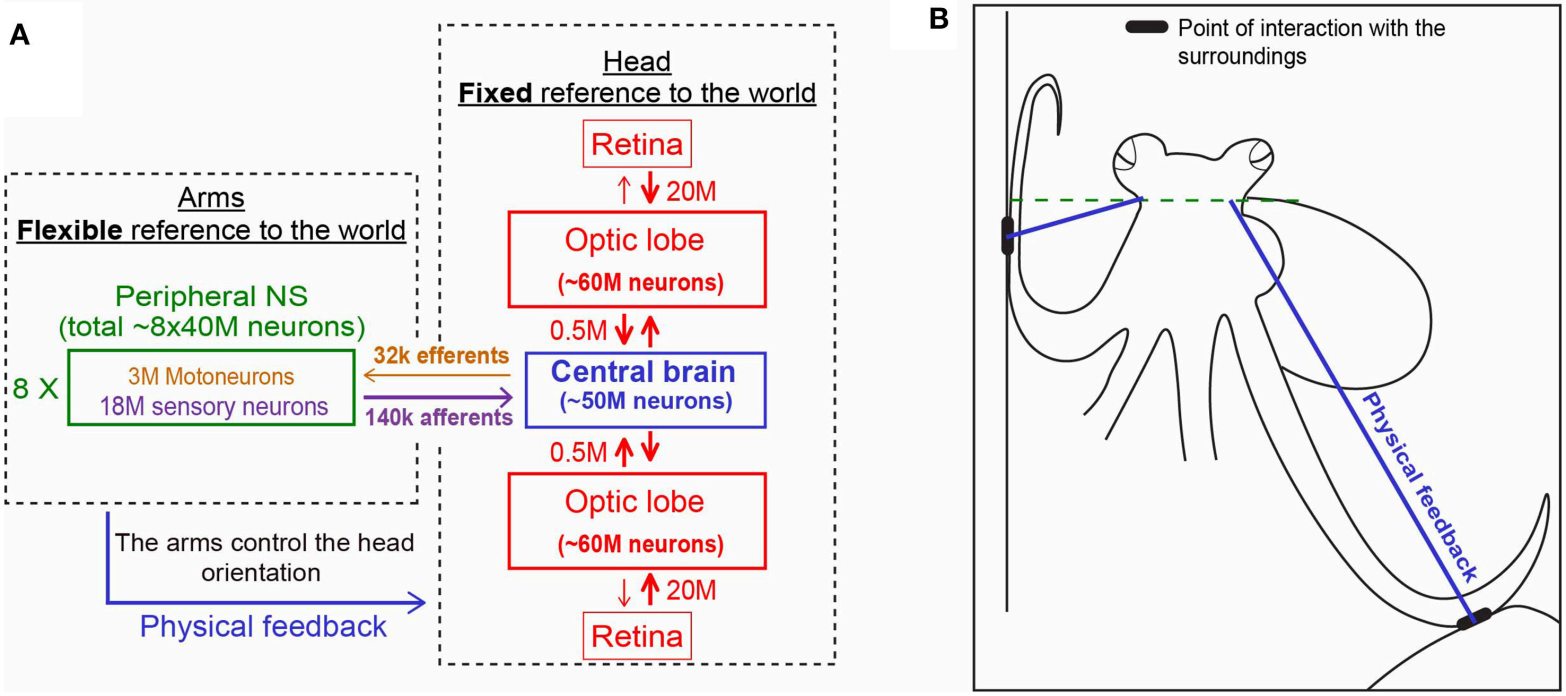
**Physical feedback from the arm to the head simplify the control of arms' interaction with the world (see main text for details). (A)** The unique distribution of the 500 million nerve cells of the octopus nervous system between its three main compartments. Each is shown in a different color. Note the relatively few fibers connecting the compartments (based on Hochner, 2012. Numbers were taken from Young, 1963, 1965). **(B)** The interaction of the arms with the sourounding provides the physical feedback that determines the fixed horizontal orientation of the head (explained schematically with the blue lines in **A,B**).

During various forms of locomotion, octopuses keep their head constantly horizontal (Figure 2 and see video). This is not surprising because, as in many animals, especially those living outside water, keeping the head in fixed reference to the external world simplifies interfacing the visual information with movement commands that drive the interaction with the external world. Indeed, even the simplest creatures have mechanisms for sensing gravity and cephalopods are known for their highly evolved vestibular system, with a pair of statocysts embedded within the rigid cartilaginous brain capsule (Barber, 1966; Young, 1971; Wells, 1978). This location of the statocysts enables them to gauge directly only the orientation of the head and thus, out of the whole soft body, to keep the head at a fixed orientation to the external world (Figure 3). This arrangement simplifies the control because the head and eyes are in a fixed reference to the external world thereby reducing the complexity involved in the interfacing of the external sensory information with the generation of motor commands needed for the interaction with the surrounding.

On the other hand, while keeping the body in a stable posture relative to the force of gravity seems fundamental and simple for animals with a rigid skeleton, it is a much more difficult challenge for an animal with flexible appendages. We find that, as in fetching, the evolved solution is based on “shaping” the soft body instead of controlling joint angles as in skeletal animals. As indicated by the name of their class “Cephalopoda,” octopus arms emerge directly from the base of the head around which they are radially distributed. During locomotion, the imaginary axis that runs between the eyes remains close to horizontal (Figure 2 and Video), implying of an active adjustment of the eyes' height by controlling the distance between the contact points of the active arms with the environment and the base of the head (Figure 3B, straight blue lines). This simplifies the controlling of the head's orientation because it is achieved by a straightforward mechanism that only controls the stiffness of the arms (Figure 3B). Such stiffens control may involve only one DOF per interacting arm. Because the octopus almost doesn't have a neck (see Wells, 1978), the horizontal visual plane of the eyes cannot move much relative to the base of the head and therefore the interaction of the arms with the environment also keeps, through the “physical feedback” (Figure 3), a stable horizontal view of the external world. If this principle is indeed implemented as the biomechanical basis of arm-propelled locomotion, it would imply that octopus locomotion is unlikely to be based on a motor program involving a robust feedforward component, as clearly apparent in locomotion of all skeletal animals which are driven by CPGs. Indeed, our kinematic analysis of octopus crawling and walking (Figure 1) suggests that both these locomotion maneuvers are controlled by what we would suggest to term a “probabilistic” strategy of moment-to-moment changes in the probability of recruiting of those arms that have the better chances of moving the body in the desired direction. Figure 1 shows that in walking (D), like crawling (C), there is no clear order in the pattern of arm recruitment. Nor does the FFT analysis of the walking velocity indicate the involvement of any CPG. Note that the lack of involvement of CPG in walking is functionally more significant than the lack of a CPG in crawling because in crawling there is no need to care for body stability as the body rests on the substrate. In walking, on the other hand, arm coordination must deal also with stability because the center of body mass is above the ground; walking control must take into consideration that at least two arms need to be in contact with external support to stabilize the body above the ground (Figure 3B).

The octopus' probabilistic control strategy, together with the radial organization of the arms around the body, creates yet another unique feature in the control of octopus locomotion. In contrast to all bilaterian animals (animals with bilateral body symmetry), the octopus can locomote in any direction relative to its facing direction and, as shown for crawling in Levy et al. (2015), at the same time it can independently control the orientation in which its body faces.

These findings further support the theory that *embodied organization* of behavior has led to the evolution of a unique body plan that enables the existence of efficient motor control mechanisms that overcome the huge complexity involved in the control of hyper-redundant soft bodied animal. In other words, the special morphology of the octopus enabled the selection of control strategies that require the nervous system to deal with a rather small number of controlled variables.

## Ethics statement

The research was conducted under the Directive2010/63/EU.

## Author contributions

GL did the research and the analysis. GL and BH designed the experiments, interpret the results and wrote the paper.

## Funding

This work was supported by European Community EP7 STIFF-FLOP project number 287728.

### Conflict of interest statement

The authors declare that the research was conducted in the absence of any commercial or financial relationships that could be construed as a potential conflict of interest.
